# Integrated probabilistic risk assessment for nanoparticles: the case of nanosilica in food

**DOI:** 10.1007/s11051-015-2911-y

**Published:** 2015-06-06

**Authors:** Rianne Jacobs, Hilko van der Voet, Cajo J. F. ter Braak

**Affiliations:** Biometris, Wageningen University and Research Centre, P.O. Box 16, 6700 AC Wageningen, The Netherlands

**Keywords:** Dose metric, E551, Margin of exposure, Uncertainty, Variability, Nanotechnology, Governance

## Abstract

Insight into risks of nanotechnology and the use of nanoparticles is an essential condition for the social acceptance and safe use of nanotechnology. One of the problems with which the risk assessment of nanoparticles is faced is the lack of data, resulting in uncertainty in the risk assessment. We attempt to quantify some of this uncertainty by expanding a previous deterministic study on nanosilica (5–200 nm) in food into a fully integrated probabilistic risk assessment. We use the integrated probabilistic risk assessment method in which statistical distributions and bootstrap methods are used to quantify uncertainty and variability in the risk assessment. Due to the large amount of uncertainty present, this probabilistic method, which separates variability from uncertainty, contributed to a better understandable risk assessment. We found that quantifying the uncertainties did not increase the perceived risk relative to the outcome of the deterministic study. We pinpointed particular aspects of the hazard characterization that contributed most to the total uncertainty in the risk assessment, suggesting that further research would benefit most from obtaining more reliable data on those aspects.

**Electronic supplementary material:**

The online version of this article (doi:10.1007/s11051-015-2911-y) contains supplementary material, which is available to authorized users.

## Introduction

Insight into risks of nanotechnology and the use of nanoparticles is an essential condition for the social acceptance and safe use of nanotechnology. Many frameworks have been developed for risk assessment of nanomaterials, as were summarized by Grieger et al. (2012). These include frameworks for risk governance, risk screening, adaptable risk assessment tools, and risk assessment and management frameworks. The majority of the frameworks were developed for environmental and occupational risk assessment.

One of the problems with which the risk assessment of nanoparticles is faced is the lack of data. This leads to uncertainties in the characteristics of nanomaterials, effects and exposure assessment, and testing considerations (Grieger et al. 2009). Specifically, within food safety, information is needed on the correct dose metric to use, the toxicokinetics of nanoparticles, the food products containing nanoparticles (Bouwmeester et al. 2009), the state of nanoparticles when manufactured and when used, and the potential for exposure (Cockburn et al. 2012). In practice, this information is hard to come by causing large uncertainty in the risk assessment (Morgan 2005). Besides the lack of data, other sources of uncertainty can include disagreement among literature sources, linguistic imprecision or uncertainty about simplifications, or models used (Morgan and Henrion 1990). Traditionally, this problem of uncertainty is solved deterministically using worst-case scenarios. For example, a worst-case scenario could make use of a highly sensitive individual, a very high concentration of the specific chemical in the product, or a very high consumption of the nano-containing foods. This method, however, compromises the transparency of the risk assessment (Jager et al. 2001) thereby leaving risk assessors groping in the dark as to the various factors contributing to the risk assessment result. Also the deterministic method does not differentiate between variability and uncertainty. There is neither quantification of uncertainty nor of variability among individual persons regarding exposure and sensitivity. An alternative is to incorporate variabilities and uncertainties into the risk assessment quantitatively using probabilistic methods. In a probabilistic risk assessment, variabilities and uncertainties can be quantified separately and the effect of all separate sources can be seen on the risk assessment.

Within the REACH framework (REACH is a legislation of the European Chemical Agency for the Registration, Evaluation, Authorization and Restriction of Chemicals) of tiered risk assessment (ECHA 2012), probabilistic risk assessment is a possible next step when deterministic risk assessment methods indicate a potential risk. Integrated probabilistic risk assessment (IPRA) was developed as a framework in which to conduct such a probabilistic risk assessment (van der Voet and Slob 2007; Bosgra et al. 2009). It was further developed and expanded to allow for more than one adverse effect (van der Voet et al. 2009). Although IPRA was developed for the risk assessment of conventional chemicals (i.e., non-nanochemicals), it is also a potential method for the risk assessment of chemicals in nano-form.

Research on the use of probabilistic methods in the risk assessment of nanoparticles in food is scarce. A Scopus search (January 28, 2015) on the keywords “probabilistic,” “risk,” “food,” and “nano,” gave 0 results. Two less restrictive searches were also performed: “probabilistic,” “risk,” and “food” (543 results) and “risk,” “food,” and “nano” (146 results). Within this apparent research void, we present a case study to illustrate the use of probabilistic risk assessment in the area of nano and food.

In this paper, we expand the deterministic study of Dekkers et al. (2011) on nanosilica in food into a fully integrated probabilistic risk assessment. In doing so, we will illustrate two points: how variability and uncertainty in a risk assessment are quantified, and how to determine which sources of uncertainty have the biggest influence on the risk assessment results.

In the “Data and method” section, we will discuss the data and methods used in our probabilistic risk assessment. The “Results” section provides the results. In the “Discussion” section, the results and limitations are discussed, followed by a short conclusion in the “Conclusion” section.

Our example is nanosilica-containing products added to food. Food additives are generally assumed safe for human consumption. E551 is a food additive known as silicon dioxide or synthetic amorphous silica (OECD 2004). It is mainly used as an anti-caking agent in powders or powder-like products such as soup powders, seasoning mix powders, and pancake mix. The characterization and physicochemical properties are extensively outlined in a JRC report (Rasmussen 2013) and summarized by van der Zande et al. (2014). According to the Federation of European Specialty Ingredient Industries, E551 does not contain nanoparticles even though they are used in the production process (ELC 2011). Dekkers et al. (2011), however, found silica in nano-form in food products that contain E551. They, therefore, performed a risk assessment of nanosilica as found in E551, describing all steps of the risk assessment process.

## Data and method

The risk assessment paradigm consists of three main parts: exposure assessment, hazard assessment (including hazard identification and hazard characterization), and risk characterization (FAO/WHO 1995). The ratio of an estimate of tolerable exposure to an exposure estimate is termed the margin of exposure (MoE). This paradigm will be used when discussing the various aspects of the deterministic and probabilistic risk assessment. To provide the background from which we develop the probabilistic risk assessment, we first discuss the deterministic risk assessment done by Dekkers et al. (2011). Next, the data used for the risk assessment are described, and then the method which makes use of the IPRA method for calculating the MoE in a probabilistic way (van der Voet and Slob 2007). Two main aspects include the use of distributions instead of worst-case values and the separation of variability from uncertainty.

### Background

A deterministic risk assessment of nanosilica in food was done by Dekkers et al. (2011). By considering labels of various brands of different powder products, 27 products were identified that contained E551. These products were measured on their silica content. In 12 of these products, the amount of nanosilica (ranging from 0 to 33 % of the total silica content) was also measured. In processed products, such as coffee with coffee creamer, this percentage was higher. The percentage nanosilica of total silica content in coffee with coffee creamer was 43 % compared with 19 % in raw coffee creamer. It was, therefore, suggested that processing increases the amount of silica in nano-form. Based on this hypothesis, a worst-case assumption of 50 % nanosilica was used for the remaining 15 products for which the amount of nanosilica was not measured. The consumption of the 27 products was based on worst-case estimates (maximal consumption) made by expert judgment. Combining the concentration with the consumption information, a worst-case exposure of 1.8 mg/kg BW/day nanosilica was obtained.

For the hazard characterization, Dekkers et al. (2011) used a published toxicity study on mice (So et al. 2008). This mouse study was a 10-week oral toxicity study with one control group and one treatment group. The treatment group was fed 1,500 mg/kg BW/day nanosilica. This study showed potential liver toxicity, which was seen in increased alanine aminotransferase (ALT) levels and fatty liver patterns after hematoxylin and eosin (H&E) staining (So et al. 2008). A lowest observed adverse effect level (LOAEL) of 1,500 mg/kg BW/day nanosilica was derived because an adverse effect was observed at the tested dose level.

In the risk characterization, Dekkers et al. (2011) estimated the ratio of the LOAEL to the estimated exposure, which we term the MoE, alternatively referred to as the margin of safety (MoS). A MoE less than one occurs when exposure is greater than LOAEL. The use of one as a threshold for safety is not appropriate, however, because the MoE compares human exposure with animal toxicity. Humans may or may not be more sensitive to a substance than animals. Traditionally, for conventional chemicals, an assessment factor of 10 is applied to animal toxicity to accommodate this difference (Lehman and Fitzhugh 1954). Besides animal to human extrapolation, we also have to deal with variability in the human population itself. A sick, young, or old human being will possibly be more sensitive to a substance than the average-aged healthy human being. This variation is usually also represented by a factor of 10 (Lehman and Fitzhugh 1954). The two assessment factors result in a combined assessment factor of 10 × 10 = 100. It is, therefore, common within the risk assessment community to compare the MoE for conventional chemicals with the tolerance value of 100: a MoE greater than 100 is deemed to be safe (Lehman and Fitzhugh 1954). In this context, the MoE (850) obtained by Dekkers et al. (2011) would be high enough for nanosilica to be judged safe. There are, however, some doubts whether this is the case. First, the scientific basis for a safety margin of 100 is unclear (ECETOC 1995). Second, whether this value is appropriate for use in the context of nanoparticles is uncertain (Dekkers et al. 2011).

There are more uncertainties in the risk assessment of nanoparticles. Another source of uncertainty is the appropriate dose metric to use. The classical dose metric used in chemical risk assessment is mass per unit of body weight. In nanoparticle risk assessment, however, this might be different. It has been suggested that for oral toxicity, particle number (N) per unit of body weight might be a more appropriate dose metric (Pasupuleti et al. 2012) and for inhalation toxicity, surface area might be a more appropriate dose metric (Maynard and Kuempel 2005). Dekkers et al. (2011) provided the risk characterization using both these dose metrics. To derive either of these dose metrics, one needs the particle size. This derivation adds uncertainty to the risk assessment because there is uncertainty about the particle size. This uncertainty results in uncertainty in the MoE. Instead of a single MoE value (850), a range of possible MoE values was obtained: 280 through 5,600 m^2^/kg BW/day and 31 through 250,000 N/kg BW/day (Dekkers et al. 2011). Here we take note that the added uncertainty of the dose metric results in a potential risk (31 is less than 100). Taking into account this source of uncertainty and other possible uncertainties such as the correct assessment factor to use and the lack of toxicity data, Dekkers et al. (2011) concluded that, even in the initial case of 850, the MoE is probably not large enough to allow for all the extrapolation steps and uncertainty. This issue is the basis for the current paper. We argue that probabilistic methods, in which uncertainty and variability are quantified as far as possible, provide a more transparent risk assessment. It decreases uncertainty about whether a certain MoE is high enough and also provides insight into which sources of uncertainty contribute most to the final risk assessment.

### Concentration data

Concentration data were obtained from the Dekkers et al. (2011) study. Of the 27 products measured for total silica content, 25 products had a positive total silica concentration. Of these 25, 11 products were also measured on nanosilica content (see Table 1). In the deterministic study, the nanosilica concentration for the 14 products not measured on nanosilica content was taken as 50 % of the total silica concentration. A recent study, however, has pointed out that this percentage is variable after consumption of the food and can even become as high as 100 % in the gastro-intestinal tract (Peters et al. 2012). This makes the measured nanosilica concentrations before consumption less relevant. We, therefore, chose not to use the nanosilica measurements, but rather the total silica measurements and model the uncertain percentage of nanosilica with a distribution. Details are explained in the “Method” section.

**Table 1:** Concentration of total silica and nanosilica in measured food products and linking to Dutch National Food Consumption Survey (DNFCS) products

Basic product	Measured products	Total silica (mg/g)	Nanosilica (mg/g)	Corresponding product types from DNFCS (percent of basic product in the DNFCS product)
Sauce powder	Mix for lasagna sauce	5.4	0.3	Sauce prepared from sauce powder (18 %)
	Cheese sauce	6.6		Dishes containing sauce (9 %)
Meat seasoning	Minced meat seasoning mix	2.6	0.2	Pure meat seasoning (100 %)
				Dishes containing meat seasoning (3 %)
Cake mix	Cake with icing	0.6		Pure cake flour (100 %)
				Cakes containing flour (55 %)
Instant noodles	Instant noodles tandoori	12.9		Noodle dishes (3 %)
	Instant noodles chicken	5.8		
Instant soup powder	Instant asparagus soup	0.6	0.2	Soup prepared from instant soup powder (6 %)
	Instant beef soup	0.6		
Coffee creamers	Coffee creamer (brand a)	5.1	1.0	Pure creamer (100 %)
	Coffee creamer (brand b)	4.9		Drinks containing creamer (3 %)
Rubs	Spicy pepper rub	1.1	<0.1	Pure bread crumbs (100 %)
	Sweets sticky rub	6.0	0.4	Dishes containing bread crumbs (10 %)
	Steak house rub	4.3	0.2	
	Roasted vegetable rub	4.9	0.6	
	Sea food rub	4.7	0.5	
International seasoning mixes	Burrito seasoning mix	7.1	0.3	Pure spice mix (100 %)
	Taco seasoning mix	11.4		Dishes containing spice mix (10 %)
	Guacamole seasoning mix	5.8		Dishes containing spice mix and a starch (5 %)
	Nasi rames seasoning mix	6.2		
Pancake mix	Pancake mix	2.8	<0.1	Pure pancake flour (100 %)
				Pancakes containing pancake flour (32 %)
Cappuccino creamer	Cappuccino foam creamer	4.9		Drinks containing cappuccino creamer (3 %)
Soy shake	Soy slim shake	3.4		Drinks prepared from soy shake (8 %)
Vitamin C tablets	Vitamin C	1.5		Vitamin C tablets, pills and capsules (100 %)
Multivitamin Junior	Multivitamins junior (brand a)	13.7		Multivitamin junior or kid tablets, pills and capsules (100 %)
	Multivitamins junior (brand b)	13.7		

Some of the 25 silica-containing products represented the same basic product, e.g., two types of instant soup powder. We identified 13 basic products with 1–5 measured products per basic product (see Table 1). The basic products are assumed to represent all the powder food types which contain the food additive E551.

### Consumption data

The consumption data were obtained from the Dutch National Food Consumption Survey (DNFCS) of 2007–2010. This survey was conducted by the National Institute for Public Health and the Environment (RIVM) from 2007 to 2010 on the Dutch population aged 7 through 69 years (van Rossum et al. 2011). It contains the consumption of 3819 respondents on two non-consecutive 24-h dietary recalls. Consumed products are identified by their NEVO code (Nederlandse Voedingsmiddelentabel, Netherlands Food Table) and classified into one of the EPIC-SOFT food (sub-)groups (Voss et al. 1998). Using these (sub-)groups, we identified products containing one or more of the measured products, i.e., all products that contain or have as ingredient a powdered product.

### Linking concentration and consumption data

The basic products had to be linked with the consumed products from the DNFCS. This linking was done by identifying products in the DNFCS, which contain or are produced from the basic products. The percentage of the basic product in the DNFCS product was determined as follows. By consulting food packaging labels, the amount of powder ingredient to produce the prepared product was determined. For complete meals that contain the prepared product, a rough estimate was made for the percentage of the meal that consists of the prepared product.

Table 1 provides a list of the basic products, corresponding measured products (with total silica and nanosilica concentrations), and corresponding product type from the DNFCS and food composition percentages. For a detailed table listing specific products from the DNFCS, the reader is referred to Table S1 in Supplementary Material.

### Dose–response data

In this paper, we use the toxicity study of van der Zande et al. (2014) as a replacement for the So et al. (2008) study used in the deterministic assessment discussed in the “Background” section. Van der Zande et al. (2014) performed a 28-day and a 84-day oral toxicity study in rats. The 84-day oral toxicity study showed increased liver fibrosis at high doses of nanosilica. This was investigated by considering ten slides of liver cells per rat. The occurrence and severity of fibrosis were noted. In the 84-day oral toxicity study, 15 rats were divided into one control and two treatment groups. In treatment 1, 5 rats were fed with synthetic amorphous silica (SAS) at an intended dose of 2,500 mg/kg BW/day. In treatment 2, 5 rats were fed with pyrogenic NM-202 [the OECD representative nanostructured silica for applications related to food (van der Zande et al. 2014)] at an intended dose of 1,000 mg/kg BW/day. After preparing the food, the realized concentration of silica in the nano-sized range (5–200 nm) was measured. The concentrations were 0 mg/kg BW/day for the control, 819 mg/kg BW/day for the SAS treatment, and 810 mg/kg BW/day for NM-202. We assumed the two treatments to be two dosages of the same substance because no or minor differences were found between SAS and NM-202 in powdered form after in vitro digestion and in intestinal solubility (van der Zande et al. 2014). In this way, we have three dose groups which we can use in a dose–response model. This is described in the “Method” section. We considered chronic risk assessment because liver toxicity is a form of chronic toxicity. To this end, we introduced a subchronic-to-chronic extrapolation factor for the toxicity study which is discussed in the “Quantifying variability in IPRA” section.

### Method

We redid the deterministic study based on new toxicity data and recent research on nanosilica fractions and performed a probabilistic risk assessment using IPRA.

In the deterministic study, we replaced the toxicity data of So et al. (2008) with that of van der Zande et al. (2014). Moreover, we replaced the 50 % nanosilica percentage by 100 % as a new worst-case estimate.

**Fig. 1:**
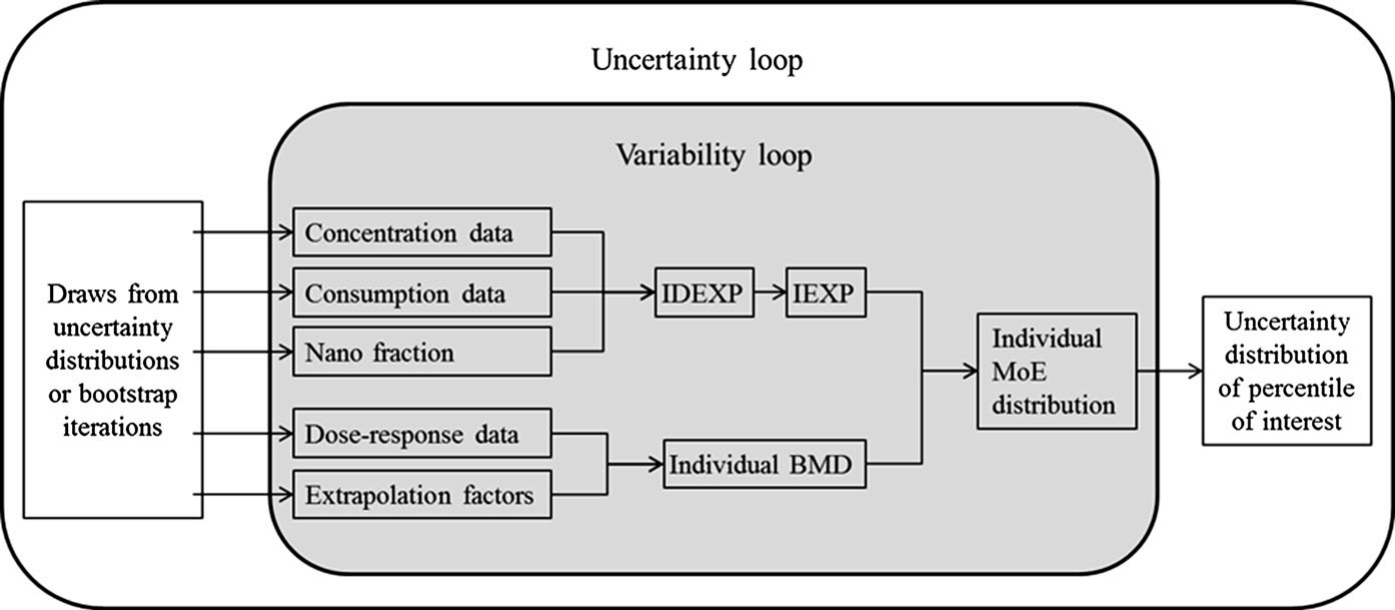
A schematic diagram of uncertainty and variability loops in the two-dimensional Monte Carlo scheme used in IPRA (*IDEXP* individual-day exposure, *IEXP* individual exposure, *BMD* benchmark dose, *MoE* margin of exposure)

IPRA uses a two-dimensional Monte Carlo scheme to quantify uncertainty and variability distributions separately in the risk assessment as illustrated in Fig. 1. Details on the various aspects of the model are discussed in the “Quantifying variability in IPRA” and “Quantifying uncertainty in IPRA” sections.

First, we describe the quantification of variability in IPRA. Second, we describe how sources of uncertainty for the nanosilica case are quantified.

#### Quantifying variability in IPRA

Following the basic three-component structure of risk assessment, we discuss exposure assessment, hazard assessment, and risk characterization as part of the IPRA method (variability loop of Fig. 1).

Exposure is probabilistically expressed as the individual-day exposure (IDEXP), which is the nanosilica intake by an individual on 1 day. The IDEXP is calculated using the formula

1 $${\text{IDEXP}} = \sum\limits_{k = 1}^{p} {{\text{CONS}}\, \times \,{\text{CONC}}_{k} }$$

with CONC_*k*_ *=* *F* × *C*_*k*_, where CONS_*k*_ is the consumption of product *k* (in g/kg BW), *C*_*k*_ is the concentration of nanosilica in product *k* (in mg/kg), and *F* is an optional factor that can be added to allow for changes in concentration and/or specific sources of uncertainty. IDEXP is then the individual exposure to nanosilica in µg/kg BW. The IDEXP distribution is obtained by calculating the IDEXP for each of the person days for which consumption data are available. This distribution represents variability in individual human intake at the person-day level. Because we consider chronic toxicity, we need long-term exposure. For this, the person-day level intake (IDEXP) distribution needs to be converted into a distribution of individual long-term exposures (IEXP). This conversion is done using the NCI model (Tooze et al. 2006), also known as the logisticnormal-normal (LNN) shrinkage model, as detailed in MCRA (2013).

The factor *F*, from now on referred to as the nanofraction, is used to convert silica concentration to nanosilica concentration. Due to the chronic nature of the risk assessment, any variability in the nanofraction will be averaged out over the long term. This means that we are only interested in a nominal nanofraction value and its uncertainty. We chose 50 % as a nominal value for the percentage of nanosilica in silica, i.e., *F* = 0.5. This nominal value was multiplied with *C*_*k*_ in Eq. (1) to produce a value for the nanosilica concentration in the basic product as found in the consumed product. The (large) uncertainty associated with *F* is discussed in the “Quantifying uncertainty in IPRA” section.

The above describes the method when using the dose metric, mg/kg BW/day. We, however, also consider the dose metric N/kg BW/day. Consumed nanosilica particles vary in diameter from 5 nm through 200 nm (Dekkers et al. 2011; Peters et al. 2012). We assume that with each consumption moment a representative sample of particle sizes is consumed. Moreover, because we consider chronic exposure, consumption moments are modeled over time, averaging out any particle size effect. This means that we are only interested in the median particle size. Considering various studies, we assumed a median particle size of 100 nm (van der Zande et al. 2014; Peters et al. 2012). This particle size is used to calculate the number of particles per mass unit (see Appendix for details). For each of the Monte Carlo iterations, the number of particles per mass unit is multiplied with the concentration, C_*k*_ of that iteration to produce an exposure value of nanosilica in 10^12^ N/kg BW/day. For convenience, we divided N by 10^12^, because N is very large.

Hazard is expressed as the individual benchmark dose (IBMD), which is the dose at which an individual human experiences a predefined response to a substance (higher IBMD means lower hazard). Starting from a BMD_animal_ obtained from a dose–response modeling of data from an animal study, the IBMD is calculated using the formula:

$${\text{IBMD}} = \frac{{{\text{BMD}}_{{{\text{animal}}}} }}{{{\text{EF}}_{{{\text{chronic}}}} \times {\text{EF}}_{{{\text{inter}}}} \times {\text{IEF}}_{{{\text{intra}}}} }}$$

where EF_chronic_ is the best estimate for an extrapolation factor for subchronic-to-chronic extrapolation, EF_inter_ is an extrapolation factor for the interspecies conversion (from the average animal to the average human), and EF_intra_ is an individual extrapolation factor for the intraspecies variation (deviation from the average human).

**Fig. 2:**
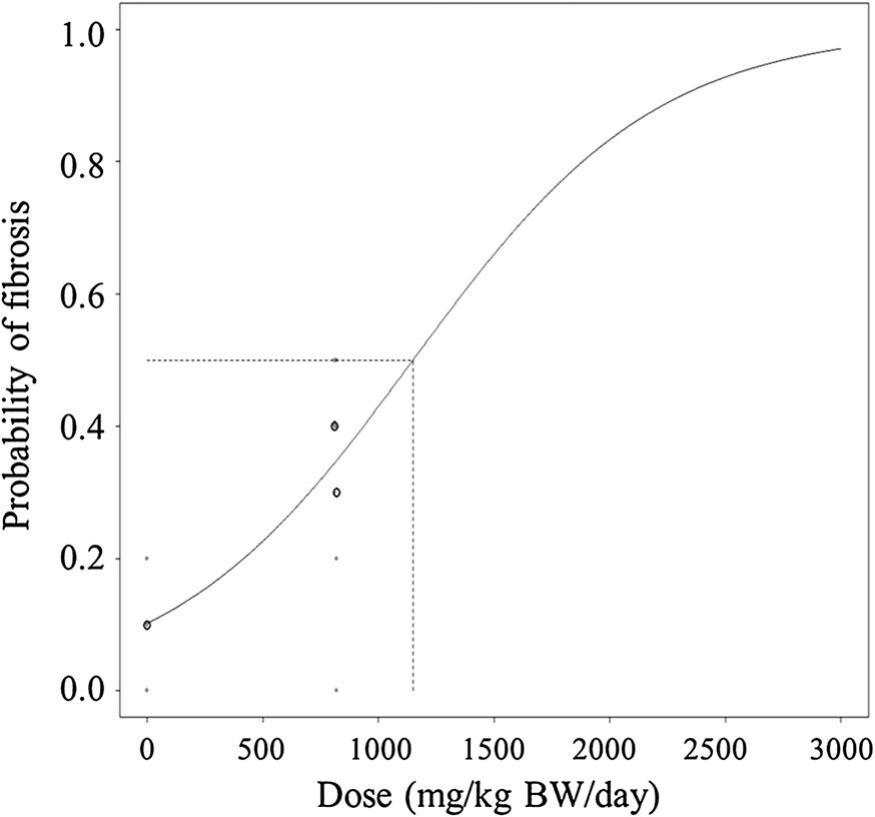
Scatterplot of the dose–response data and fitted logistic regression model for dosages of 0, 810, and 819 mg/kg BW/day of nanosilica. The response is the probability of having a positive (as defined by van der Zande et al. 2014) fibrosis slide out of ten liver cell slides. The *vertical dashed line* indicates the BMD. The *circles* indicate the mean response of each dose group

To obtain the BMD_animal_, we modeled the dose–response data using PROAST software (Slob 2002; Slob and Cotton 2013). The effect which we used to quantify the toxicity of nanosilica was the number of positive fibrosis slides (out of 10) per rat. This effect variable has a Binomial (10, p) distribution for each rat, with *p* the probability of obtaining a positive fibrosis slide for that rat. We modeled this probability using seven different models: logistic, probit, log-logistic, log-probit, weibull, gamma, and linearized two stage (Barlow et al. 2009). All seven models passed the goodness of fit test at a 5 % level of significance. For each model, PROAST calculated a BMD as the ED_50_, which is the only relevant statistic from a dose–response curve on quantal data when the variation around the ED50 does not represent true inter-personal differences in response but only differences between laboratory animals and other measurement errors (Slob et al. 2011, 2014). We also calculated the Akaike information criterion (AIC) for each model to determine the best model. From Table 2, we see that the logistic model is the best fitting model (i.e., lowest AIC), although the differences are small. From this model (Fig. 2), we obtained the nominal BMD_animal_ of 1,160 mg/kg BW/day. Model uncertainty is discussed in the next section.

**Table 2:** Dose-response models with calculated log-likelihood, AIC, and BMD

Model	Number of parameters	Log-likelihood	AIC	BMD
Null	2	−86.99	177.98	NA
Full	4	−80.45	168.9	NA
Linearized two-stage	4	−81.03	170.06	1300
Log-logistic	4	−81.02	170.04	1630
Weibull	4	−81.03	170.06	1330
Log-probit	4	−81.01	170.02	2650
Gamma	4	−81.02	170.04	1500
Logistic	3	−81.05	168.1	1160
Probit	3	−81.06	168.12	1450

The null and full models are added for comparison

*NA* not applicable

The EF_chronic_ accounts for the extrapolation from a subchronic study to a chronic risk. Bokkers and Slob (2005) studied such extrapolation based on the No-observed-adverse-effect level (NOAEL) and the benchmark approach. Not all studies which derive a NOAEL, however, are suitable for benchmark modeling. A more recent study, therefore, only used the NOAEL approach (Batke et al. 2011). We, therefore, used the data collected by Batke et al. (2011) to obtain a median EF_chronic_ of 1.475.

The EF_inter_ accounts for the extrapolation from the average animal to the average human. Interspecies differences can be quantified in different ways, such as extrapolation based on body weight, caloric demand, or surface area (Vermeire et al. 1999). We used caloric demand because it is a “biologically plausible and data-based extrapolation method applicable to a wide range of chemical substances” (Schneider et al. 2004) and is preferred above body weight scaling (Vermeire et al. 1999). In allometric body weight scaling based on caloric demand, the ratio of animal to human dose is equal to the ratio of animal body weight to human body weight raised to the power 0.75 (Vermeire et al. 1999). The EF_inter_ based on the test species used, in our case the rat, is obtained as

$${\text{EF}}_{\text{inter}} = \frac{{{\text{dose}}\,{\text{rate}}_{\text{rat}} }}{{{\text{dose}}\,{\text{rate}}_{\text{human}} }} = \,\frac{{{\text{dose}}_{\text{rat}} /{\text{bw}}_{\text{rat}} }}{{{\text{dose}}_{\text{human}} /{\text{bw}}_{\text{human}} }} = \frac{{{\text{dose}}_{\text{rat}} }}{{{\text{dose}}_{\text{human}} }}\frac{{{\text{bw}}_{\text{human}} }}{{{\text{bw}}_{\text{rat}} }} = \,\left( {\frac{{{\text{bw}}_{\text{rat}} }}{{{\text{bw}}_{\text{human}} }}} \right)^{0.75} \frac{{{\text{bw}}_{\text{human}} }}{{{\text{bw}}_{\text{rat}} }} = \left( {\frac{{{\text{bw}}_{\text{human}} }}{{{\text{bw}}_{\text{rat}} }}} \right)^{0.25} = \,\left( {\frac{70}{0.25}} \right)^{0.25} \, \approx \,4.$$

We used the average body weight values as given by Vermeire et al. (1999).

The IEF_intra_ accounts for variability that exists within the human population. We obtained the distribution describing this variability using the method of van der Voet et al. (2009) and the assumption that the 95th percentile sensitive person is 2–10 times more sensitive than the average person (the range describing uncertainty). The variability distribution is obtained as a log-normal distribution with geometric mean equal to one and a geometric standard deviation of 1.91 by simultaneously accounting for the variability and the uncertainty (see the “Quantifying uncertainty in IPRA” section for further details).

To convert the dose metric of the IBMD, the number of particles per mass unit as obtained previously was multiplied by the BMD of 1160 to obtain a BMD in 10^12^ N/kg BW/day.

Finally, in the risk characterization part, we obtain the distribution of the individual margin of exposure (IMoE) by combining independent draws of IBMD and IEXP

$${\text{IMoE}} = \,\frac{\text{IBMD}}{\text{IEXP}}.$$

A person is at risk when his/her exposure is greater than his/her critical effect dose. Hence, an individual is at risk when IMoE <1.

#### Quantifying uncertainty in IPRA

The method explained above is a probabilistic risk assessment that accounts for variability that is present in the human population (the variability, inner, loop of Fig. 1). The next step is to account for uncertainty (outer loop of Fig. 1). Again, we will consider exposure and hazard separately.

For exposure, we need to consider the consumption data, concentration data, and nanofraction. Sampling uncertainty in consumption data was quantified by bootstrapping the data at the level of individual persons (500 iterations). We quantified the uncertainty in the concentration data as far as possible by bootstrapping the repeated measurements per basic product. Due to the small number of measurements, however, this probably underestimates the uncertainty. This limitation is further discussed in the “Limitations” section.

Uncertainty in the nanofraction, *F*, was quantified by a statistical distribution. As mentioned in the “Concentration data” section, according to recent research, the nanofraction, *F*, can be up to one (100 %). This uncertainty about the nanofraction is modeled by a logistic-normal distribution such that its 50th percentile (p50) is equal to 0.5 and its 95th percentile (p95) is equal to 0.8. The logistic-normal distribution was chosen because resulting values of *F* are fractions bounded by 0 < *F* < 1. The probability density function of a logistic-normal distribution is given by

$$f(x) = \,\frac{1}{{\sigma \sqrt {2\pi } }}\,\exp \,\left( { - \frac{{({\text{logit}}\,(x) - \mu )^2}}{{2\sigma^{2} }}} \right)\frac{1}{x(1 - x)},$$

where 0 < *x* < 1 and ${\text{logit}}\,(x) = \ln \left( {\frac{x}{1 - x}} \right)$ (Aitchison and Shen 1980). This distribution is denoted by *F* ~logisticnormal (μ, σ). Considering various distributional shapes (see Fig. S1 in Supplementary Material), we chose p95 = 0.8. This allows for at least 1 % chance of values greater than 0.85 and at least 1 % chance of values less than 0.15.

In each uncertainty iteration, an IEXP distribution was calculated using inputs from the bootstrapped data and uncertainty distributions. In this way, uncertainty in the IEXP distribution was quantified.

For hazard, we need to consider uncertainty in the BMD_animal_, BMD_chronic_, EF_inter_, and IEF_intra_. We first consider the BMD_animal_. The BMD_animal_ is subject to two main sources of uncertainty: limitations of the dose–response data and model uncertainty. These uncertainties were quantified by generating 100 datasets from each of the seven dose–response models (see previous section) by the parametric bootstrap method (Efron and Tibshirani 1994, p 53; Moerbeek et al. 2004). From these, the BMDs were calculated, and all 700 BMD values pooled into one set. This set now includes uncertainties from the limitations of the dose–response data and model uncertainty.

The uncertainty in the EF_chronic_. was quantified by a log-normal distribution with expected value 1.80 and standard deviation 1.52 (Batke et al. 2011).

From the “Quantifying variability in IPRA” section, we calculated the nominal EF_inter_ as four. This value, however, is uncertain due to uncertainty about potential toxicokinetic and toxicodynamic differences between rats and humans. This uncertainty was accounted for by deriving an uncertainty distribution around EF_inter._ This derivation was done assuming the 99th percentile (p99) of the uncertainty distribution to equal 10 (Slob and Pieters 1998). With four as the geometric mean of the log-normal distribution, a geometric standard deviation (1.48) for the uncertainty distribution was obtained.

The IEF_intra_ was assumed to contain both variability and uncertainty. The variability distribution was already given in the “Quantifying variability in IPRA” section. The uncertainty in the geometric standard deviation was quantified by a $\chi^{2}$-distribution. By setting the 2.5th percentile (p2.5) of the uncertainty distribution equal to 2 and the 97.5 percentile (p97.5) equal to 10, we are able to solve for the number of degrees of freedom for the $\chi^{2}$-distribution (van der Voet et al. 2009).

By drawing an uncertainty value for each of the BMD_animal_, EF_chronic_, EF_inter_, and IEF_intra_, we calculated the IBMD. Repeating this process for 500 uncertainty iterations, we quantified the uncertainty in the IBMD distribution. In the risk characterization, independent draws from the uncertainty distributions of the IEXP and the IBMD were combined into the IMoE. In this way, uncertainty in the IMoE distribution was quantified from which the uncertainty distribution of percentiles was obtained.

A simple graphical representation of both variability and uncertainty of the IMoE can be given in the form of a so-called IMoE bar graph. In an IMoE bar, a box represents the variability distribution of the IMoE between specified percentiles. These can be more or less extreme percentiles (denoted by px for the xth percentile), e.g., p0.1 and p99.9, p1 and p99, or p5 and p95, depending on the level of protection required. Whiskers are used to represent one-sided uncertainty of these percentiles. We chose uncertainty limits such that the left whisker represents the lower 5 % uncertainty bound of the lower percentile and the right whisker represents the upper 95 % uncertainty bound of the upper percentile.

In addition to considering the effect of uncertainty on the final risk assessment, it is important to identify the extent to which sources of uncertainty contribute to the total uncertainty present in a certain percentile of interest. In IPRA, a probabilistic sensitivity analysis was implemented to this effect, following the method of van der Voet and Slob (2007). In short, seven sources of uncertainty were considered, namely consumption data, concentration data, nanofraction, BMD, subchronic-to-chronic factor, interspecies factor, and intraspecies factor. These seven sources of uncertainty result in a full 2^7^ factorial design where sampling from the uncertainty distribution for each source is turned on and off. For each of the uncertainty runs, 2^7^ = 128 values are obtained for a given percentile. In this way, 128 distributions are obtained each of which is summarized by its variance. An additive model is then fitted to the 128 variances. When this model explains most of the variance, which is usually the case, the coefficients of the main effects can indicate the contribution to the total variance. The intercept term estimates uncertainty when all specified seven sources of uncertainty are turned off and represents uncertainty due to Monte Carlo calculations. Results are illustrated by means of a bar graph, showing the percentage contribution of each source of uncertainty.

## Results

**Fig. 3:**
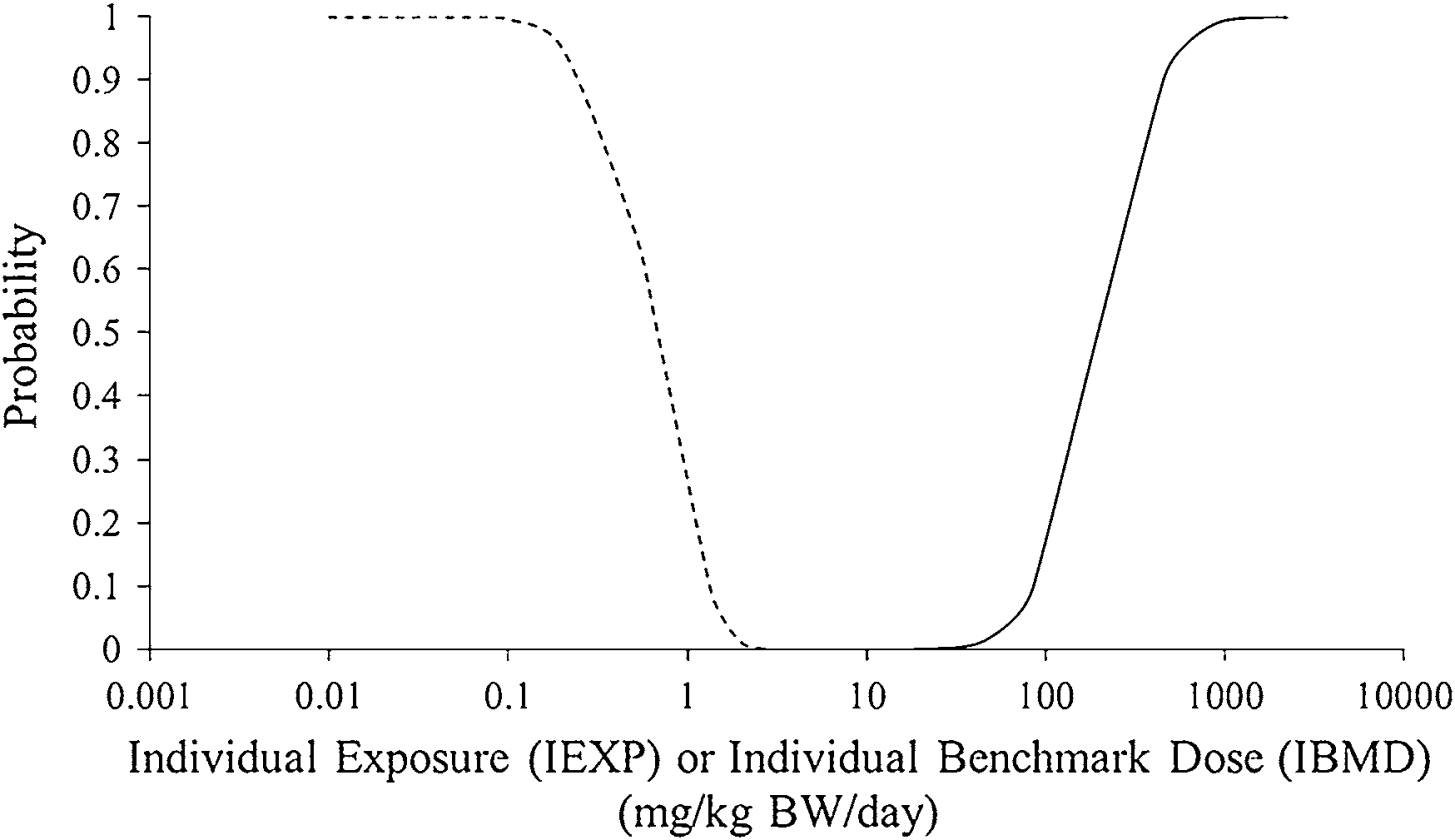
The IEXP exceedance distribution (*dashed curve*) and IBMD cumulative distribution (*solid curve*). The amount of overlap of the *curves* indicates the amount of risk present

Figure 3 illustrates the IEXP and the IBMD distributions. The IEXP distribution is plotted as an exceedance curve indicating the percentage of the population that exceeds the exposure value on the *x* axis. The IEXP curve starts at 1, indicating that all individuals have some intake of nanosilica on the long run. This is due to the nature of a chronic risk assessment. The amount of overlap of the curves, which indicates the amount of risk present and is related to the expected risk concept of van Straalen (2002), appears small.

**Fig. 4:**
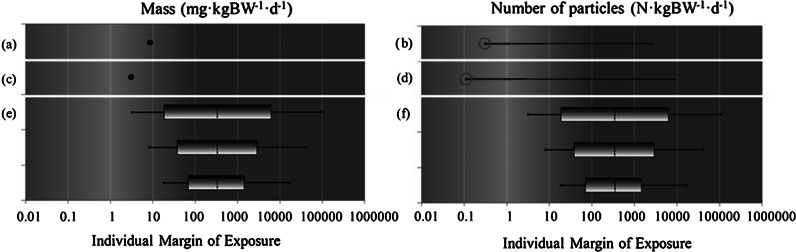
Deterministic estimates MoE^*^ = MoE/100 in mg/kg BW/day (*a*, *c*) and in N/kg BW/day (*b*, *d*) according to Dekkers et al. (2011) (*a*, *b*) and calculated with more recent data (*c*, *d*) and IMoE bars illustrating the variability and uncertainty distributions of the IMoE (*e*, *f*). The *three boxes* in each *plot* represent IMoE distributions p0.1–p99.9, p1–p99, and p5–p95, respectively, in mg/kg BW/day (*e*) and in N/kg BW/day (*f*). In each *box*, the *left* *whisker* represents the lower 5 % uncertainty bound of the lower percentile. The *right* *whisker* represents the upper 95 % uncertainty bound of the upper percentile. The *dashed line* indicates the median of the IMoE variability distribution. *Background coloring* visualizes the transition from high (*red*) to low risk (*green*). (Color figure online)

Figure 4 shows the results of the deterministic and probabilistic risk assessments. Figure 4a–d provides the deterministic MoE^*^ values. Note that the deterministic MoE values were divided by 100 to make them comparable with the IPRA results (MoE^*^ = MoE/100). The values of MoE^*^ calculated according to Dekkers et al. (2011) are shown in Fig. 4a (MoE^*^ = 8.5) and Fig. 4b [MoE^*^ = (0.31–2,500)], whereas the values of MoE^*^ calculated using the more recent data as detailed in the “Method” section are shown in Fig. 4c (MoE^*^ = 2.94) and Fig. 4d [MoE^*^ = (0.11–8736.36)]. As discussed in the “Data and method” section, the MoE^*^ when using mg/kg BW/day seems to be on the safe side. The lower limits of the MoE^*^ when using N/kg BW/day, however, give some cause for concern, because they are less than one.

 Figure 4e provides the probabilistic equivalent of Fig. 4c and f that of Fig. 4d. IMoE bars are shown below one another for p0.1–p99.9, p1–p99, and p5–p95. Changing to a probabilistic approach including quantified uncertainties does not lead to a higher perceived risk, but rather tends to confirm that the results from the deterministic study are worst case. Moreover, considering the uncertainty in particle size in a probabilistic way has removed the concern of possible risk in the deterministic case. Comparing Fig. 4e with 4f we see that the different dose metrics give comparable results, in contrast to the deterministic study. This difference between the deterministic study and our probabilistic study is due to the fact that in the deterministic study, all extremes in particle size were included in the calculations, while in the probabilistic study, consumers are expected to be confronted with the whole distribution of particle sizes, and therefore the particle size effect cancels out in a chronic risk assessment.

**Fig. 5:**
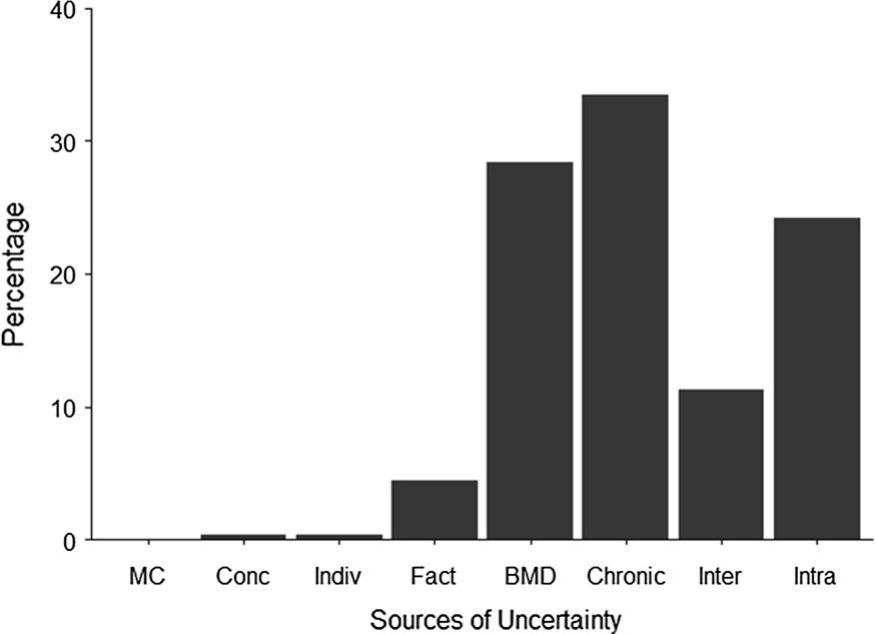
A *bar* graph illustrating the relative contribution of each source of uncertainty to the total uncertainty of the 1st percentile (p1) of the IMoE distribution. The sources of uncertainty are *MC* Monte Carlo uncertainty, *Conc* concentration uncertainty, *Indiv* consumption uncertainty, *Fact* nano fraction (*F*) uncertainty, *BMD* BMD_animal_ uncertainty, *Chronic* EF_chronic_ uncertainty, *Inter* EF_inter_ uncertainty, and *Intra* IEF_intra_ uncertainty. The variance explained by the additive model is 99.38 %

For any choice of percentile of the IMoE distribution as the main indicator of a possible risk, it is important to determine which sources of uncertainty influence its value. As an example we will consider p1, which is the left side of the middle box in the IMoE bars in Fig. 4e and f. Figure 5 illustrates the contribution of each source of uncertainty to the total uncertainty of the p1 of the variability distribution of the IMoE. The highest contributions to uncertainty of p1 are the BMD, subchronic-to-chronic extrapolation factor, and intraspecies factor, together accounting for more than 80 % of the total uncertainty. A similar pattern was obtained for p0.1 and p5 (not shown).

## Discussion

One of the main features of IPRA is its ability to quantify uncertainty and variability separately. This is important because uncertainty is in principle reducible, whereas variability is not. Uncertainty is variation existing due to lack of data or information. In this paper, the quantified uncertainty gave better insight about whether the MoE obtained from the deterministic study was large enough, given the large uncertainties. We conclude that this is the case, because our results indicate that even with the uncertainty accounted for, we still end up on the safe side. We cannot claim, however, that nanosilica in food poses no risk, because certain uncertainties were not quantified. This is discussed in the next subsection.

A feature of IPRA is the ability to study the contribution of each source of uncertainty to the total uncertainty. Knowledge of the most influential sources of uncertainty can focus research to those areas in order to decrease this uncertainty. We found that uncertainty in the BMD, the subchronic-to-chronic extrapolation factor, and the intraspecies factor contributed most to the total uncertainty in the risk assessment. Better dose–response data and more research on dose–response model choice, subchronic-to-chronic extrapolation, and on the variability of human sensitivity would contribute much to reducing the total uncertainty.

To decrease the uncertainty in the identified sources, more research is needed to obtain more data and better understanding of the data. More research in the form of experiments can be expensive. An additional method for obtaining data is expert elicitation (Linkov et al. 2009). In such a method, experts are asked to give their opinion on a certain variable. These opinions can be used to obtain a first impression about the uncertainty of that variable. This prior knowledge can then be used to improve on the uncertainty distributions which are fitted to the variables in a probabilistic risk assessment, possibly in a Bayesian context. One such an approach was illustrated in the development of a multi-criteria decision model based on expert judgment (Flari et al. 2011).

### Limitations

Although we replaced many worst-case assumptions where possible with statistical distributions, we still made worst-case assumptions in some instances. These include the decisions on which consumed products contain the measured products and how much of the measured product do they contain. The description of products in the DNFCS and the description of measured products are not detailed enough to be able to make perfect matches. We, therefore, assumed that all DNFCS products mentioned in Table S1 (see Supplementary Material) were prepared using the basic product mentioned in the first column. This assumption, of course, is a worst-case assumption because DNFCS products could well have been alternatively prepared or readymade. On the other hand, it is possible that there are other food products that also contain E551. In that case, we could have underestimated exposure. For some food composition percentages, we assumed a worst-case scenario. For instance, dishes containing sauce were assumed to consist 50 % of sauce (e.g., minced meat and white sauce).

The worst-case scenarios could have been replaced by distributions describing the uncertainty. This could be done by including more factors in Eq. (1) to quantify the various uncertainties. These factors would then represent uncertainty about whether we included all products that contain E551, about the food composition percentages and about the increase of nanosilica in processed products.

Another concern could be the choice of 100 nm for the median particle size. This choice was based on studies (van der Zande et al. 2014; Peters et al. 2012) in which particle sizes were measured. Due to the limitations of the instrumentation and the difficulty in measuring single particles in a large agglomeration such as found in food products, a perfect estimate for the median particle size was not possible. Further research might increase our knowledge.

The contribution of the uncertainty of the nano fraction is directly related to the uncertainty distribution chosen. The wider this distribution, the more uncertainty we provide. Our choice was explained in the “Quantifying uncertainty in IPRA” section, but other choices are possible. Our results, therefore, should be interpreted with this in mind. Choosing a different value for p50 and p95 could have resulted in a different situation, especially in the sensitivity analysis as illustrated in Fig. 5.

It should be noted that not all sources of uncertainty were quantified. The uncertainty in silica concentration was not well quantified because of the very limited number of measurements (only 1–5 measurements per food product). Another unquantified uncertainty is whether the SAS and NM-202 used in the toxicity study are comparable to the nanosilica found in food for human consumption. A recent study questioned the use of SAS for risk assessment of nanosilica in food (Dekkers et al. 2013). Further unquantified uncertainties could be related to limited toxicity data (only one study with two dose groups), technical limitations in measuring nanosilica concentrations in toxicity studies and exposure assessment, and possible interaction with other (nano) substances which could increase or decrease the toxic effect of nanosilica. More research is, therefore, necessary to find and reduce other uncertainties that were not quantified. We suggest that a probabilistic risk assessment be part of a larger risk assessment framework in which possible other aspects of risk or unquantifiable uncertainties are dealt with qualitatively.

Finally, there are limitations to the application of current risk assessment methods to nanoparticles. A case study on nanosilver questioned whether the REACH requirement of demonstrating ‘safe use’ of a substance is currently possible for nanomaterials (Pronk et al. 2009). To support a full risk assessment for regulatory needs, further research is necessary in generating high-quality data and developing methodologies (Peters 2011).

## Conclusion

In this paper, we expanded the deterministic risk assessment of Dekkers et al. (2011) into a fully integrated probabilistic risk assessment. The overall result of the probabilistic analysis of the risk of nanosilica in food products shows, with 95 % confidence, that at least 99 % of the Dutch population would experience no risk. This conclusion is similar for the two metrics chosen, in contrast to the results of Dekkers et al. (2011) who indicated a much lower possible MoE on the particles scale. We do need to caution that this risk assessment is not 100 % comprehensive and should only be seen as an illustrative exercise. The results are not intended as an authoritative risk assessment on nanosilica.

Approaching this risk assessment from a probabilistic method, we obtained a more transparent picture of risks and a better insight into the extent that uncertainty plays in the risk assessment process. We, therefore, conclude that in cases where deterministic methods show possible risks in a lower-tier assessment and different sources of variability and uncertainty are known to influence the results, probabilistic methods of risk assessment are preferable.

### Electronic supplementary material

Supplementary material 1 (PDF 165 kb)

## Appendix

- In this section, we detail the calculations for obtaining a different dose metric. To convert mg/kg BW/day to number of particles, we use the particle diameter and the density of nanosilica, assuming that particles are solid spheres.
- 1. Calculate the volume of one particle (in nm^3^):
- $$\nu_{\text{particle}} = \,\frac{4}{3}\pi r^{3}$$
- with *r* the radius of a particle in nm.
- 2. Calculate the mass of one particle (in mg):
- $$m_{\text{particle}} = \,\nu_{\text{particle}} \times \,d_{\text{nanosilica}}$$
- with $d_{\text{nanosilica}}$ the density of nanosilica in mg/nm^3^.
- 3. Calculate the total number of particles per mg:
- $$N_{\text{mg}} = \,\frac{1}{{m_{\text{particle}} }}.$$
