# Supplementary material for: Integrated probabilistic risk assessment for nanoparticles: the case of nanosilica in food
Source: J Nanopart Res. 2015 Jun 6;17(6):251. doi: 10.1007/s11051-015-2911-y (PMC4457916; doi:10.1007/s11051-015-2911-y)

Supplementary material

Integrated probabilistic risk assessment for nanoparticles: the case of nanosilica in food
Journal of Nanoparticle Research

Rianne Jacobs (corresponding author)
Biometris
Wageningen University and Research Centre
P.O. Box 100
6700 AC Wageningen
The Netherlands
Tel: 00 31 (0) 317 483574
E-mail: rianne.jacobs@wur.nl

Hilko van der Voet
Wageningen University and Research Centre
Wageningen, The Netherlands

Cajo J.F. ter Braak
Wageningen University and Research Centre
Wageningen, The Netherlands

Table S1: Linking of measured food products and DNFCS food products

Basic product	Measured product	%	Corresponding product(s) from DNFCS
Sauce powder	Mix for lasagna sauce<br>Cheese Sauce	18<br><br><br><br><br><br><br><br>9	Sauce: cocktail, béarnaise, butter, Vietnamese, veloute, white, bacon, onion, ragout, peanut, “ravigotte”, tandoori, tomato with vegetables, herbs, tomato plain, sweet pepper, wrap, Thai, Italian, hollandaise, for potatoes bacon/onion, pepper-cream, minced meat roll, non-specified, oyster, for vegetables based milk/maizena, schaschlik, roux, for potatoes herbs/garlic, Chicken Tonight Hawaii, sweet/sour exotic, for meals, for “wereldgerecht”, hoisin, babi-pangang, pepper, broth, red wine, white wine, vegetables, sweet pepper-cream, stroganoff, Piccalilli, “hunting”, ginger, mushrooms, pizza, ham-cheese, curry, cream, cheese, Hawaii, “wereldgerecht” minced meat and green beans, tuna, filled wine, filled curry, bolognaise, tahini, spaghetti, pasta, paprika, macaroni, vegetable, minced meat, pilav,<br>Dishes containing sauce: lasagna with tomato, minced meat and white sauce, spaghetteria, chicken fillet in ketjap sauce, spaghetti carbonara, macaroni with braised vegetables, lasagna bolognaise, chicken in curry sauce, spaghetti bolognaise, macaroni with ham and cheese, lasagna with spinach, cheese and eggs, chicken with sweet-sour pineapple sauce, macaroni dish, Lai chi kai, chicken in wine sauce, lasagna, cheese sauce soufflé, lasangette napolitana, macaroni bake
Meat seasoning	Minced meat seasoning mix	100<br>3	Maggi mix for oven dishes,<br>meatball, peppers stuffed with minced meat, cabbage roll stuffed with minced meat, meatball prepared with bread, meatball prepared with sponge-cake, meatball stuffed with egg, macaroni with minced meat and braised vegetables, cottage pie, minced meat, canned minced meat, small meatballs, meat loaf, minced meat leek dish, meatrolls, meatdish, ready-made meatball, burgers non-specified, vegetarian minced meat, sauerkraut with minced meat, beef/pork minced meat, lasagna with tomato minced meat and white sauce, pork minced meat, stuffed brinjal with minced meat, Pikanto minced meat, stuffed tomato with minced meat, Hamburger, pressed minced meat, minced meat with vegetables, roasted/boiled minced meat, roasted minced meat, ground beef, minced lamb, “wereldgerecht” minced meat/greenbeans sauce, Swedish bullar minced meat, ground meat non-specified
Cake mix	Cake with icing	100<br>55	cake flour, flour non-specified,<br>iced cake, “Glacekoek”, Pimm’s cake, cake filled with cream and fruits, rolled cake, cake with sweetener, chocolate cake, cake with macaroons, apple cake with sweetener, cake with nuts, pastry non-specified, cakes non-specified, cream-filled cake, mocha sponge cake, ordinary cake, whole wheat cake, cake with cherries, marbled cake, apple cake, raisin cake, iced cake, sponge cake with cream, wholemeal apple cake, eggnog cake, bavarois cake, chipolata cake, marzipan cake, pastries with cream non-specified, meringue cake, schwarzwalder torte, fruit cake non-specified, butter cake, butter cake with nuts, sponge, chocolate muffin, muffin non-specified, muffin with fruit, lardy cake, lardy cake with sugar icing, almond cake, “Appelrondo”, Sachertorte, sponge cake with fruit
Instant noodles	Instant noodles tandoori<br>Instant noodles chicken	3	noodles, noodles dish
Instant soup powder	Instant asparagus soup<br>Instant beef soup	6	non-specified, clear with meat and vermicelli, clear with meat, vermicelli and vegetables, with legumes and meat, clear with meat, clear with vermicelli and vegetables, with legumes without meat, clear with vermicelli, clear with vegetables, thick with meat and vegetables, thick with meat, clear with meat and vegetables, thick with

			vegetables, Noodle, peanut, creamy smooth, clear with fish, Turkish yoghurt soup, pumpkin, thick with fish
Coffee creamers	Coffee creamer (brand a)<br>Coffee creamer (brand b)	100<br>3	creamer,<br>chicory, coffee with caffeine, instant coffee instant flavored, espresso n.s., espresso without caffeine, powdered thickened milk for coffee, cereal coffee, pacha nuts coffees, liquid coffee, espresso with caffeine, pacha coffee, bambu coffee, coffee with milk from machine, coffee with caffeine, coffee with 50% caffeine, coffee without caffeine, coffee with sugar and milk from machine, coffee without caffeine
Rubs	Spicy pepper rub<br>Sweets sticky rub<br>Steak house rub<br>Roasted vegetable rub<br>Sea food rub	100<br>10	bread crumbs with chicken spices, bread crumbs, mix with spices/herbs for dressing, mix with spices/herbs for cold table sauce, mix with spices/herbs for dip, mix<br>seasoning for pasta, mix seasoning for omelet/chili, mix marinade, seasoning mix, schnitzel with mushrooms + breadcrumbs, Turkey schnitzel, Valess fillet in crumbs, pork sate schnitzel with breadcrumbs, fish fillet in crumbs, fish products in crumbs, Valess mini's in crumbs, schnitzel with cheese and breadcrumbs, fish schnitzel, bread crumbs with minced meat spices, pork schnitzel, beef schnitzel, chicken schnitzel, veal schnitzel, vegetarian schnitzel, schnitzel with breadcrumbs, chicken sate schnitzel with breadcrumbs, schnitzel non-specified
International seasoning mixes	Burrito seasoning mix<br>Taco seasoning mix<br>Guacamole seasoning mix<br>Nasi rames seasoning mix	100<br>10<br>5	spice mix with vegetables for nasi/bami/etc., seasoning mix for "wereldgerecht", mix seasoning for nasi/bami/etc.,<br>siam chicken without rice, tandoori chicken without rice,<br>babi pangang, bami with crab and prawns, tandoori chicken with rice, bami, tjap tjoy with chicken, siam chicken with rice, burrito's, Mexican taco, nasi with egg, Mexican potato bake, Mexico melange, bami prepared from sachet, babi ketjap, Mexican minced meat bake, tjap tjoy with pork, Mexican meat snack, "Bamibal", nasi, Taco shell, mexican burger, "Nasibal", bami with egg, bami without egg, babi pangang, nasi goreng with prawns, mexican rice, wrap grilled mexican twister, nasi without egg
Pancake mix	Pancake mix	100<br>32	several grains pancake flour, pancake flour non-specified, plain pancake flour,<br>pancakes with ginger, pancakes with pineapple, crepes, pancakes with apple, pancake several grains, pancakes with raisins, pancakes with ham, pancakes with cheese, pancake non-specified, normal pancakes, wholemeal pancake
Cappuccino creamer	Cappuccino foam creamer	3	instant cappuccino with caffeine, cappuccino non-specified, instant cappuccino without caffeine, instant cappuccino non-specified, cappuccino with caffeine, cappuccino without caffeine
Soy shake	Soy slim shake	8	soy drink
Vitamin C tablets	Vitamin C	100	Vitamine C Kruidvat C60 sinaasappelsmaak, Vitamine C Trekpleister, Vitamine C AOV (poeder), Vitamine C Dagravit vanille-citroensmaak, Vitamine C Etos C-1000, Vitamine C Dagravit, Vitamine C Previte, Vitamine C Orthica Dino-C , Vitamine C/zink capsules EuRho, Vitamine C Lidl Vitafit lemon zonder suiker, Vitamine C Solgar 1000 mg, Vitamine C Now Foods, Vitamine C Etos 60 mg suikervrij, Vitamine C Roter Vitamine C-bruis 1000 mg, Vitamine C VitaTotaal C-1000 mg (Aldi), Vitamine C De Tuinen vitamine C 500 mg kauwtabl, Vitamine C Hema C-1000, Vitamine C Nutrilite Acerola C, Vitamine C Roter, Vitamine C Forte Weerstand kauw, Vitamine C Roter Vitamine C 120 mg vitagums, Vitamine C Albert Heijn C-1000, Vitamine C De Tuinen C-1000 mg, Vitamine C Erica C-1000 TR Rose-hip, Vitamine C supplement n.s., Vitamine C Now C-1000 with rose hips, Vitamine C Essential Organics 500 mg, Vitamine C Essential Organics 1000 mg, Vitamine C Etos C-250, Vitamine C ActionFit C-50, Vitamine C AOV C 1000mg/bioflav. 50mg, Vitamine C Artelle C-1000 met 200

			mg citrus, Vitamine C Kruidvat Vitamine C-1000 mg (bruis), Vitamine C Nova Vitae C 1000 mg, Vitamine C Phital Vitamine C Forte 1000 mg, Vitamine C HEMA C-60 met sinassmaak<br>Vitamine C Roter Blaas, Vitamine C Sunwell C 1000 mg (bruis), Vitamine C Toppharm C 1000 mg met bioflavonoiden
Multivitamin Junior	Multivitamins junior (brand a)<br>Multivitamins junior (brand b)	100	Multivit./min. Centrum Junior, Multivit./min. DA Multi voor kinderen framboos, Multivit./min. Dagravit Junior 30, Multivit./min. Davitamon Junior kauw/dragee, Multivit./min. Etos Multi Kind Alles in 1, Multivit./min. Hema Kind Multi Totaal (kauw), Multivit./min. Multi Gummies for Kids, Multivit./min. Optimax kinder, Multivitamine Kruidvat Multi-vit. voor kinderen, Multivit./min. Kruidvat Multi A-Z kinderen, Multivit./min. Samenw. Apoth. voor kinderen, Multivit./min. Lucovitaal Multi+ Kids omega 3-6 sv, Multivit./min. Bloem Kind&Balans, Multivit./min. Etos Multi Alles in 1 Kind kauw, Multivit./min. NatuFit Multi Junior, Multivit./min. De Tuinen Multi Kind, Multivitamine A.G.Natural Health Junior, Multivitamine Kinder Multivitaminen Gezond & Wel Multivitamine ActionFit Kinder Multi, Multivit./min. Dagravit Kids-Xtra 6-12 jaar, Multivit./min. Vitastar Kindermulti, Multivitamine Roter vitagums-opgr. Kinderen, Multivit./min. Flinndal Multi Junior, Multivit./min. Orthomol Immun Junior

Fig. S1 Histograms of nano-fraction (factor) for various parameter choices

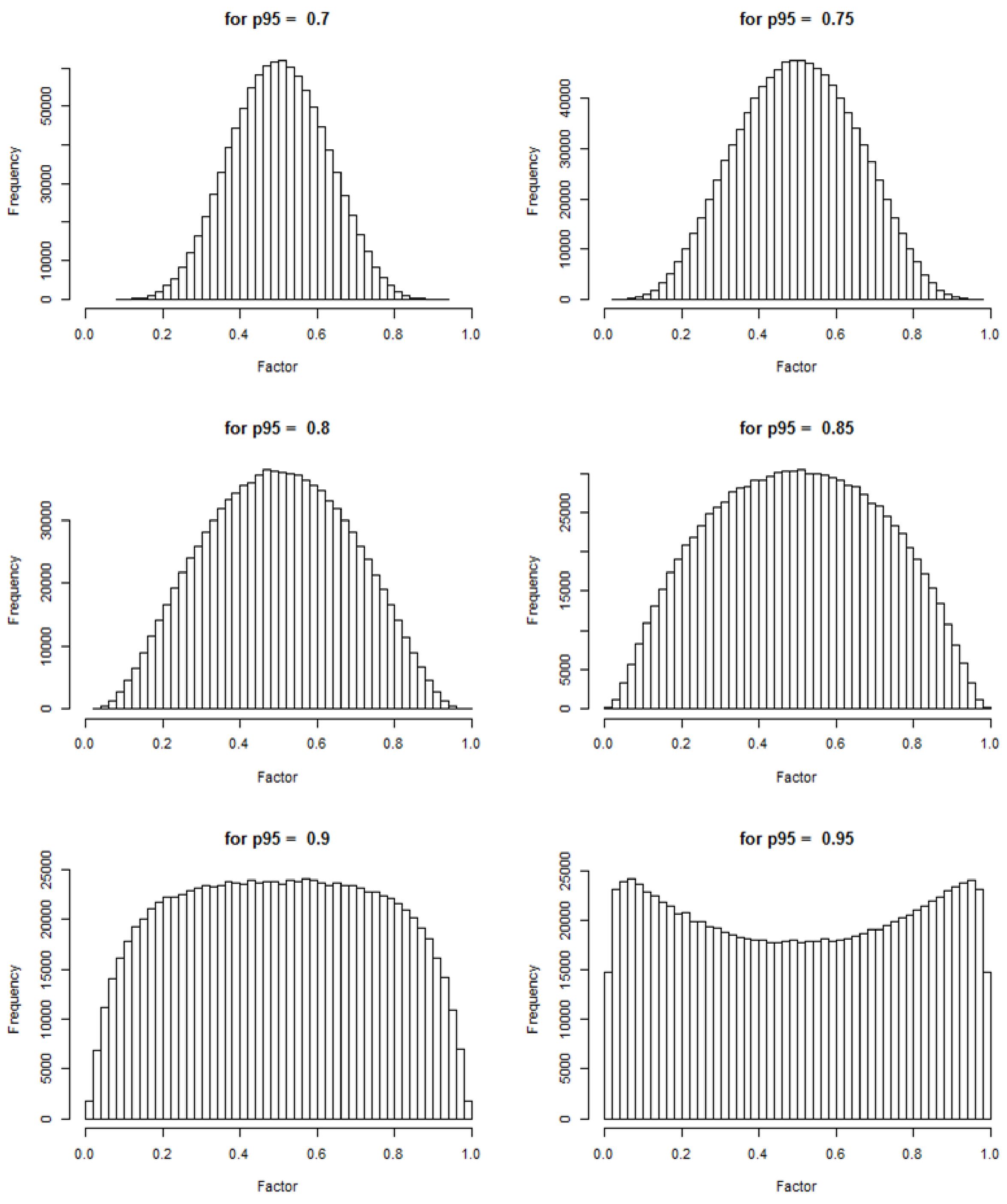

Supplement: Supplementary file 1 — Supplementary material 1 (PDF 165 kb) [file 11051_2015_2911_MOESM1_ESM.pdf]
